# Targeting liposarcoma: unveiling molecular pathways and therapeutic opportunities

**DOI:** 10.3389/fonc.2024.1484027

**Published:** 2024-12-11

**Authors:** Hongliang Liu, Xi Wang, Lingyan Liu, Bingsong Yan, Fabo Qiu, Bin Zhou

**Affiliations:** ^1^ Department of Hepatobiliary and Pancreatic Surgery & Retroperitoneal Tumor Surgery, The Affiliated Hospital of Qingdao University, Qingdao, China; ^2^ Department of Oncology, Women and Children’s Hospital Affiliated to Qingdao University, Qingdao, China; ^3^ Department of Gastroenterology, Qilu Hospital of Shandong University, Jinan, China; ^4^ Department of Hepatobiliary Surgery, Women and Children’s Hospital Affiliated to Qingdao University, Qingdao, China

**Keywords:** liposarcoma, driver genes, molecular pathogenesis, targeted therapy, outlook

## Abstract

In recent years, an increasing number of studies have utilized molecular biology techniques to reveal important molecular heterogeneity among different subtypes of liposarcoma. Each subtype exhibits distinct genetic patterns and molecular pathways, which may serve as important targets for molecular therapy. In the present review, we focus on the molecular characteristics, molecular diagnostics, driver genes, and molecular mechanisms of liposarcoma. We also discuss the clinical research progress of related targeted therapies, with an aim to provide a reference and crucial insights for colleagues in the field.

## 1 Introduction

Liposarcoma (LPS) is the most common soft tissue sarcoma (STS) in adults, accounting for 15%–20% of STS, and can also occur in adolescents and children. It is a malignant tumor derived from adipose cell differentiation (1). According to the fifth edition of the World Health Organization Classification of Tumors of Soft Tissue and Bone, published in 2020 (2), the LPS subtypes comprise atypical lipomatous tumor (ATL)/well-differentiated LPS (WDLPS), dedifferentiated LPS (DDLPS), myxoid LPS (MLPS), pleomorphic LPS (PLPS), and myxoid pleomorphic LPS. The main treatment for all LPS subtypes is surgical resection; however, for patients with unresectable, advanced, or metastatic LPS, treatment options are currently limited and often ineffective, resulting in a generally poor prognosis. New drugs are therefore urgently needed to improve the current state of treatment. In recent years, the continuous development of molecular biology techniques has resulted in the stratification of genetic subgroups within LPS. Concurrently, an increasing number of clinical and research-oriented treatments have been tested based on an understanding of the specific molecular pathology of each subtype; these studies have yielded good progress and results. In the present review, we discuss the molecular characteristics, molecular diagnostics, driver genes, and molecular pathogenesis of LPS. We also explore the corresponding therapeutic targets and downstream pathways, and summarize progress toward targeted therapies for several subtypes of LPS.

## 2 Molecular characteristics of LPS

The LPS subtypes differ in their clinical behaviors, treatment sensitivities, and underlying biological characteristics. In the following sections, the detailed molecular characteristics of each subtype are described in terms of genomics, proteomics, and epigenetics.

### 2.1 Genomics

The different STS subtypes exhibit molecular heterogeneity. Nacev et al. identified specific somatic mutations and copy number alterations in some subtypes via the genetic sequencing of STS samples, and compared tumor mutational burden and microsatellite instability across the different subtypes (3). STS can be divided into two genomic categories. One category (80%) consists of tumors with complex karyotypes, such as leiomyosarcoma, undifferentiated pleomorphic sarcoma, DDLPS, and anigosarcoma. These tumors are characterized by many gene rearrangements and chromosomal gains or losses that often include cell cycle–related genes such as *TP53*, *MDM2*, *RB1*, and *CDK4* (4). The other category (20%) consists of tumors with specific genetic alterations, such as gene translocations and activating point mutations (5). Importantly, tumors with specific genetic alterations can also develop complex karyotypes as the tumor progresses. Taylor et al. reported that different subtypes of retroperitoneal liposarcoma (RPL) have distinct genomic landscapes, and discussed the genomic differences between RPL and extremity LPS (6).

WDLPS and DDLPS share several common genetic features. Research by Wagner and his team indicates that WDLPS and DDLPS evolve from common precursors into distinct patterns (7). The molecular signatures of both subtypes are characterized by amplifications in the 12q13-15 region on the long arm of chromosome 12 (8). Molecular testing indicates that approximately 90% of WDLPS/DDLPS cases have confirmable *MDM2* and *CDK4* gene amplifications, which are the primary driver genes (9). In recent years, an increasing number of whole-genome sequencing studies have identified that additional gene amplifications within the 12q13-15 region in WDLPS/DDLPS (such as the amplification of *HMGA2*, *TSPAN31*, *FRS2*, *GLI1*, *YEATS4*, *YEATS2*, *NAV3*, and *CPM* in WDLPS), new genes outside the 12q13-15 region (such as *DDR2* and *SDHC* in WDLPS, and *FGFR3* in DDLPS), and receptor tyrosine kinase (RTK)-related signaling pathways are closely associated with the occurrence and progression of WDLPS/DDLPS (10–12).

Given the shared genetic characteristics between DDLPS and WDLPS, and the observation of both well-differentiated and poorly differentiated areas in many DDLPS samples, DDLPS is commonly believed to evolve from WDLPS. However, these two sarcomas differ substantially. During dedifferentiation, ongoing DNA damage leads to genomic instability and the further accumulation of complex genomic aberrations. In DDLPS, pathways related to cell proliferation and the DNA damage response are upregulated, whereas in WDLPS, pathways related to adipocyte differentiation and metabolism are upregulated (13). Studies have also reported that the loss of 11q23 and the amplification of 6q23 or 1q32 (or the co-amplification of 6q23 and 1q32) are DDLPS-specific genomic abnormalities. Additionally, intrachromosomal and interchromosomal gene rearrangements and gene fusions (such as *C15orf7*::*CBX3*, *CTDSP1*::*DNM3OS*, and *CTDSP2*::*DNM30S*) have been identified in DDLPS but not in WDLPS. *DDIT3* is also amplified in DDLPS patients (14). Furthermore, a study that comprehensively analyzed the molecular characteristics of retroperitoneal sarcoma-WDLPS revealed that *FOXD4L3* has periodic mutations that interact with the PAX pathway to promote tumorigenesis. Moreover, the phosphoinositide 3-kinase (PI3K)/protein kinase B (AKT) and mitogen-activated protein kinase (MAPK) pathways, as well as genes associated with the transition from an adipose to a “tumor” phenotype, are all dysregulated (15). Pollock et al. reported that Aurora A kinase (AURKA) is significantly overexpressed in retroperitoneal sarcoma-DDLPS and is strongly associated with metastasis and recurrence (16). Combined, large-scale whole-exome and RNA sequencing in Japan has revealed that somatic copy number alterations are the most common genomic mutations in DDLPS (17). The frequency of mutations varies for each chromosome, ranging from 0.114 (chromosome 21) to 0.482 (chromosome 12). In this study, DDLPS was then divided into the following three groups based on the associations between somatic copy number alterations and clinical features: cluster 1, with only 12q15 high magnification; cluster 2, with 12q15 and 1p32.1 high magnification; and cluster 3, without 12q15 high magnification. A survival analysis conducted after the genomic clustering revealed that, compared with cluster 1 patients, cluster 2 DDLPS patients had better progression-free survival (PFS) rates. Multivariate regression analysis revealed that cluster 1 was a significant predictor of poor PFS, independent of the surgical margin and primary tumor site. Furthermore, a comparative analysis of WDLPS and DDLPS components revealed that the gene sets associated with cell cycle progression, including the G2/M checkpoint and *E2F* target genes, were significantly enriched in DDLPS. By contrast, a gene set associated with adipocyte differentiation or lipid metabolism, including adipogenesis and fatty acid metabolism, was significantly enriched in WDLPS.

Lago et al. reported that DNA G-quadruplexes (G4s) in the promoters of lipopolysaccharide-treated cells are associated with high transcription levels in open chromatin, indicating that promoter G4s and related transcription factors work in concert to form cell-specific transcriptional programs (18). Moreover, Richter et al. reported that mouse double minute 2 homolog (MDM2) induces the formation of stable G-quadruplexes, which are specifically recognized by cellular helicases. The targeting of G-quadruplexes can reduce MDM2 expression and p53 degradation, thereby promoting cell cycle arrest and apoptosis in cancer cells (19).

MLPS is genetically characterized by the translocation of t(12;16)(q13;p11) in more than 95% of cases; this results in the *FUS*-*DDIT3* fusion gene, which stimulates cell proliferation and disrupts adipogenic differentiation (20). The remaining 5% of MLPS cases are genetically characterized by the translocation of t(12;22)(q13;q12), which results in the *EWSR1*-*DDIT3* fusion gene (20). These features are considered unique to MLPS. Additionally, high *RET* expression has been observed in MLPS, and approximately 25% of all cases have mutations that activate the PI3K/AKT/mammalian target of rapamycin (mTOR) signaling pathway (21). Moreover, over 50% of MLPS cases carry mutations in the *TERT* promoter (22).

PLPS is characterized by marked chromosomal abnormalities, including chromosomal deletions and duplications (23). Although related molecular research is limited, studies have reported that mutations or inactivation of *RB1* are associated with PLPS development (24). Furthermore, genetic testing of a metastatic lesion in a patient with uterine PLPS with liver metastasis revealed an *IQGAP*-*NTRK3* gene fusion (25).

Myxoid pleomorphic LPS exhibits complex chromosomal changes; however, it lacks the *FUS*-*DDIT3* gene fusion that is characteristic of MLPS and the *MDM2*/*CDK4* gene amplification found in DDLPS (2). Molecular research in this area is also limited.

### 2.2 Proteomics

Proteomic technologies and strategies are increasingly being applied to the study of STS. Huang et al. conducted proteomic analyses of different STS subtypes. By mining the proteomic data of cluster of differentiation 3 (CD3)^+^ tumor-infiltrating lymphocyte (TIL) groups in patients with DDLPS, these authors revealed that the high CD3^+^ TIL group was enriched in aspects such as T-cell activation, T-cell receptor signaling, leukocyte proliferation, cell adhesion, and the interferon response. By contrast, the low-CD3^+^ TIL group was enriched in the complement cascade, with an active complement system. These findings support the future evaluation of combination therapy with anti-programmed cell death protein 1 (PD-1)/programmed death-ligand 1 (PD-L1) checkpoint inhibitors and complement inhibitors to treat DDLPS patients in the low CD3^+^ TIL group (26, 27). Moreover, the data from this study suggest that, at the protein level, cyclin-dependent kinase 4 (CDK4) is expressed at a relatively high level in DDLPS. This finding is consistent with the amplification of *CDK4* in many DDLPS, although no enriched ontology was observed in an overexpression analysis of DDLPS. It has also been reported that vesicular trafficking proteins are an independent prognostic factor for distant metastasis. In addition, through the joint analysis of proteomic and phosphorylation data, a team led by Ding demonstrated STS subtypes with different molecular characteristics and clinical outcomes, and identified the key driving molecules for STS metastasis and proliferation (28). Fat metabolism-related pathways, peroxisome proliferator–activated receptor (PPAR) pathways, and vitamin metabolism pathways are significantly upregulated in DDLPS and MLPS. Furthermore, the PI3K/AKT/mTOR signaling pathway was significantly upregulated in DDLPS. Numerous molecular markers associated with pathological subtypes were also validated, including CDK4 in DDLPS. Notably, this study conducted an integrated analysis of histopathological subtypes, a hierarchical clustering of pathological subtypes, a proteomic analysis of subtypes, and an analysis of immune subtypes. The findings revealed the relationships between STS subtypes under different classification criteria, as well as their respective molecular, pathway, and clinical characteristics. In this integrated analysis, a detailed division of STS was noted, and STS heterogeneity was explored in great detail.

Together, these findings indicate that LPS has extensive molecular heterogeneity. Further exploration and discoveries of molecular differences and unique molecular characteristics will provide a wide range of ideas and directions for the experimental design and treatment of LPS.

## 3 Driver genes and molecular mechanisms

The generation of different LPS subtypes is caused by their relatively unique driver genes and molecular mechanisms, which ultimately lead to large differences between subtypes. In the following sections, the main driver genes and molecular pathways of each LPS subtype are described.

### 3.1 Molecular mechanisms related to WDLPS/DDLPS

#### 3.1.1 Molecular mechanisms associated with MDM2 amplification in WDLPS/DDLPS

The most important function of MDM2 is to control p53 activity, by acting as a negative regulator of p53 (29). MDM2 amplification is mutually exclusive with p53 gene mutation; when MDM2 is amplified, p53 is not mutated, and only wild-type p53 is present (30). The cellular tumor antigen p53 (TP53) pathway in cancer cells can be reactivated by inhibiting MDM2−TP53 interactions, thereby inducing apoptosis and inhibiting tumorigenesis.

MDM2 may also promote tumor growth through other mechanisms. Cyclin-dependent kinase inhibitor 2A (CDKN2A, also known as P14ARF or p16INK4a) is a tumor suppressor protein encoded by *CDKN2A*, which is overexpressed in WDLPS/DDLPS. CDKN2A causes MDM2 to be localized in the nucleolus, thus preventing TP53 degradation (31). Furthermore, MDM2 regulates serine metabolism and redox homeostasis independently of TP53 to drive tumor growth, and targeting the function of MDM2 in serine metabolism can inhibit DDLPS growth (32). Chen et al. reported that panhistone deacetylase 2 (HDAC2) is co-expressed with MDM2 in DDLPS, and that specific targeting of HDAC2 can reduce the expression of MDM2, which plays a role in antitumor activity (33).

#### 3.1.2 Molecular mechanisms associated with CDK4 amplification in WDLPS/DDLPS

CDK4 plays a role in LPS progression by negatively regulating the retinoblastoma protein (Rb) signaling pathway. However, CDK4 can also promote tumor growth through mechanisms that are independent of the Rb pathway. For example, CDKN2A overexpression can inhibit the Rb pathway–dependent function of CDK4 (34).

#### 3.1.3 Role of the fibroblast growth factor/FGF receptor signaling pathway in LPS

In LPS, studies have identified activating mutations, amplifications, and the overexpression of genes related to the FGFR pathway (35–37). FGFR1 and FGFR4 overexpression is observed in approximately 30% of DDLPS cases and is associated with a poor prognosis (38). In approximately 90% of DDLPS cases, *FRS2* is coamplified with *MDM2* and plays a role in tumor progression. Additionally, FGFR2 is overexpressed in MLS, where it regulates cell proliferation, apoptosis, and migration (39).

#### 3.1.4 Possible molecular mechanisms of dedifferentiation

Although up to 10% of WDLPS can progress to DDLPS, molecular research on the progression from WDLPS to DDLPS remains limited. Amplification events, such as c-Jun amplification during dedifferentiation, play a role in the occurrence and development of LPS. In DDLPS, transcription factor Jun (JUN) and apoptosis signal-regulating kinase 1 (ASK1)/mitogen-activated protein kinase 5 (MAP3K5) are coamplified; these are located in the regions of chromosomes 6q23 and 1p32. By contrast, these changes have never been reported in WDLPS. JUN amplification is strongly associated with DDLPS, although it is also observed in some cases of ATL and WDLPS. Approximately 91% of DDLPS cases express c-Jun, whereas its amplification or expression is rare in pure WDLPS. Both the *JUN* and *ASK1*/*MAP3K5* products are involved in the c-Jun N-terminal kinase (JNK) signaling pathway. *JUN* encodes a protein that regulates the activity of transcription-related factors in adipocytes, and *ASK1* encodes a kinase that activates the JNK pathway, leading to JUN activation. *JUN* or *ASK1* amplification suggests that the dedifferentiation of WDLPS ultimately leads to changes in the tissue type and the development of DDLPS (10).

In a study of exome and transcriptome sequencing data from 17 patients diagnosed with both WDLPS and DDLPS, DDLPS samples generally had a slightly greater mutational burden than matched WDLPS samples; however, this apparent difference did not reach significance. When the overall differences in gene expression between WDLPS and DDLPS samples were compared, 357 genes were highly expressed in WDLPS tumors compared with DDLPS tumors; *FABP4*, *ADIPOQ*, *LPL*, *LEP*, and *PTGER3* had the highest gene expression. The 395 genes that were less highly expressed in WDLPS tumors included the genes that were upregulated in DDLPS. In addition, among the known markers of adipocyte differentiation, *PPPARG*, *CEBPB*, *CEBPD*, *FOXO1*, *FOXO3*, *FOS*, *JUN*, *MYC*, and *CDKN1A* were also expressed at higher levels in WDLPS than in DDLPS.

In nine frozen pairs of WDLPS and DDLPS samples, 933 gene fusion transcripts were identified, with a median of 39 fusion transcripts per sample. Notably, the number of fusions in DDLPS samples was significantly greater than that in matched WDLPS samples. In DDLPS samples, only 17% of fusions were shared with homologous WDLPS samples, on average. This finding suggests that, after detachment from the clonal origin, new chromosomes in DDLPS tumors may experience more break-fusions than those in WDLPS tumors. *HMGA2* and *CPM* fusions on Chr12q occurred more frequently and were more prevalent in DDLPS samples than in WDLPS samples. In addition, *HMGA2* was significantly overexpressed in DDLPS samples. Shared somatic mutations indicated the clonal origin of matched WDLPS and DDLPS tumors, with early differentiation and genomic instability caused by the continued production and selection of new chromosomes. The random generation and expression of fusion transcripts of new chromosomes, such as *HMGA2* and *CPM*, may influence subsequent tumor differentiation status (40).

The amplification of genes located at chromosome 12q13-15 differs significantly between WDLPS and DDLPS, and may be related to progression and dedifferentiation. Amplification of the following genes in the 12q region was confined primarily to DDLPS: *MAP3K12*, *TBX5*, *CDK2*, *GLI1*, and *ALX1*. Moreover, DDLPS had a significantly higher average amplification rate than WDLPS. A key component of dedifferentiation is the loss or downregulation of adipogenesis, which leads to the formation of nonadipogenic masses that are histologically indistinguishable. Various genes are involved in fat cell metabolism. Some of these genes, including *PLIN*, *PLIN2*, and *LIPE*, are uniquely absent in DDLPS, suggesting that these cells have lost their ability to function as fat.

Bouzid et al. reported that *HMGA2* amplification is significantly associated with ATL/WDLPS but not DDLPS (10). Furthermore, Wood et al. speculated that several potential parallel signaling pathways may be involved in the dedifferentiation process of WDLPS/DDLPS (41). The Wnt signaling pathway reportedly inhibits preadipocyte differentiation (42). Moreover, Wnt signaling plays an important role in LPS occurrence and development (11). The Wnt antagonist Frzb reduces c-Met expression and inhibits Met-mediated signaling, which may be a new therapeutic strategy for STS (43). MiR-193b targets the Hippo signaling effector YAP1 to indirectly inhibit Wnt/β-catenin signaling, resulting in the inhibition of LPS cells (44). Hedgehog signaling is also involved in the regulation of adipogenesis, with one study suggesting that the aberrant activation of Hedgehog signaling during adipose tissue development leads to myogenic cell–derived rhabdomyosarcoma (45). Gli is reported to be commonly coamplified with *MDM2* and *CDK4*, and Gli-mediated upregulation of the Hedgehog signaling pathway is enriched in dedifferentiated adipose progenitor cells and DDLPS tumor cells, resulting in undesirable immune cell infiltration of the tumor (46). Notch signaling also plays a role in the adipocyte differentiation process. A recent study reported that Notch signaling activation is associated with DDLPS occurrence through the inhibition of lipid metabolism (47).

Notably, a synthetic PPAR-γ ligand reverses DDLPS dedifferentiation and blocks LPS formation. Moreover, activation of the autophagy and phosphatase and tensin homolog (PTEN)/PI3K/AKT/mTOR pathways can inhibit Notch signaling, thereby promoting the adipogenic differentiation of mesenchymal stem cells (48). Activation of the Notch/platelet-derived growth factor receptor beta (PDGFRβ) signaling pathway can also inhibit the differentiation of brown adipose progenitor cells in mice (49). Furthermore, the synthesis of PPAR-γ ligands reverses DDLPS cell dedifferentiation and prevents LPS formation (47).

#### 3.1.5 Changes in microRNA expression in WDLPS/DDLPS

The differential expression of multiple miRNAs has been identified in WDLPS/DDLPS and may have an important effect on WDLPS/DDLPS growth. In one study, more than 40 dysregulated miRNAs were identified in DDLPS, and restoring the expression of downregulated miR-143 inhibited DDLPS cell proliferation and induced apoptosis (50). In another study, compared with normal adipose tissue, miR-155 expression was upregulated in all LPS subtypes except WDLPS, and the knockdown of overexpressed miR-155 inhibited DDLPS proliferation and growth (51). A later study revealed 35 miRNAs (four with high expression and 31 with low expression) that were able to distinguish between WDLPS/DDLPS and normal fat (52). The targeting of these aberrantly expressed miRNAs may have therapeutic potential for patients with WDLPS/DDLPS; however, their exact roles and mechanisms of action in WDLPS/DDLPS remain to be clarified.

### 3.2 Preclinical research advances in determining the molecular mechanisms of MLS

#### 3.2.1 Role of the Hippo/YAP1 pathway in MLS

Hartmann et al. reported that MLS occurrence and development depend on the Hippo/YAP1 pathway, and that the *FUS*-*DDIT3*-driven tyrosine-protein kinase receptor (IGF-IR)/PI3K/AKT signaling pathway promotes the stability and nuclear accumulation of YAP1 by “turning off” the Hippo signal. *FUS*-*DDIT3* and YAP1/TEAD colocalize in mesenchymal stem cells and MLS cells to jointly regulate proliferation, cell cycle progression, apoptosis, and adipogenic differentiation (53, 54). Moreover, an increasing body of research emphasizes the importance of dysregulated Hippo signaling in MLS (55).

#### 3.2.2 Key functional interactants of *FUS*-*DDIT3* in chromatin remodeling complexes in MLS

Nelson et al. reported that several members of chromatin remodeling complexes, including NuRD (Nucleosome Remodeling and Deacetylase) and SWI/SNF (SWItch/Sucrose NonFermenting), are present in the *FUS*-*DDIT3* interactome and play key roles in regulating genomic structure and gene expression (56). Kadoch et al. confirmed that, in MLS, *FUS*-*DDIT3* inhibits the targeting and activity of the BAF complex, thereby suppressing DNA accessibility and failing to activate the target gene *CEBPB* (an adipogenic transcription factor), which ultimately reduces adipogenesis (57). Additionally, *FUS*-*DDIT3* activates the SRC/focal adhesion kinase (FAK)/RHOA/C GTPases (RHO)/Rho-associated coiled-coil-containing protein kinases (ROCK) signaling axis in MLS to increase the invasive capacity of MLS cells (58).

#### 3.2.3 Others

The bromodomain and extraterminal domain (BET) family is a group of epigenetic regulatory proteins that can modulate gene expression and are involved in tumor occurrence and development. Chen et al. reported that BET proteins promote core transcriptional regulatory programs in DDLPS (59). Furthermore, Xu et al. reported that the absence of MAPK-interacting serine/threonine protein kinases 1 and 2 (MNK1/2) inhibits STS occurrence (60).

### 3.3 Roles of long noncoding RNAs in LPS

Kirtonia et al. reported that many oncogenic long noncoding RNAs, including *MALAT1*, *PVT1*, *SNHG15*, *LINC00152*, and *MIR210HG*, are differentially expressed in LPS (61). Similarly, Yuhong et al. reported that *LINC00423* expression is downregulated in retroperitoneal sarcoma; this is primarily caused by the disruption of *NFATC3* stability, thus activating the MAPK signaling pathway (62).

### 3.4 Interaction of extracellular vesicles in the tumor microenvironment of LPS

Cancer-derived extracellular vesicles facilitate intercellular communication and transport bioactive molecules within the tumor microenvironment to impact tumor occurrence, progression, and metastasis. In RPL-DDLPS, extracellular vesicles carrying “cargo” MDM2 are released into the microenvironment, and *MDM2* DNA from RPLPS is transferred to target recipient cells—preadipocytes—in the tumor microenvironment. This transfer leads to impaired p53 activity and increased matrix metalloproteinase 2 (MMP2) production in preadipocytes, which is involved in tumor cell dissemination and recurrence (63, 64).

### 3.5 Cancer stem cells

#### 3.5.1 Notch signaling in tumor-initiating LPS cells

Shihua et al. enriched tumor-initiating cells to obtain cells with sustained Notch activation (mLPS1) and cells with normal Notch activity (mLPS2). When transplanted into mice, only mLPS1 gave rise to LPS; these cells highly expressed tumor stem cell markers (CD133) and mesenchymal stem cell markers (CD73, CD90, CD105, and Delta-like homolog 1 [DLK1]). Moreover, the clustered regularly interspaced short palindromic repeats (CRISPR)-mediated destruction of Notch signaling inhibited mLPS1 tumorigenicity (65).

#### 3.5.2 Role of the Janus kinase/Signal transducer and activator of transcription signaling pathway in cancer stem cells in MLS

Steinberg et al. reported that a subpopulation of MLS cells with cancer stem cell characteristics possess an activated JAK/STAT signaling pathway, which controls and monitors the number of cells with cancer stem cell properties (66).

#### 3.5.3 Role of the PIK3R3/Extracellular signal-regulated kinase/Nanog signaling pathway in sarcoma stem-like DDLPS cells

Yoon et al. reported that the PIK3R3/ERK/Nanog signaling pathway promotes the cancer stem cell phenotype in DDLPS, and identified PIK3R3 as a potential therapeutic target for DDLPS. In addition, Nanog knockdown and AKT inhibition can reduce the formation of spheroid cells and reverse drug resistance to doxorubicin and radiation (67, 68).

To date, progress in research into driver genes and molecular pathways has elucidated the mechanisms of LPS in a stepwise manner. These studies have also provided insights and guidance regarding the content and direction of clinical research.

## 4 Molecular targeted therapies

Genes and their expression products, related molecular pathways, and intermolecular interactions all play important roles in LPS occurrence and development. On the basis of these findings, the corresponding possible therapeutic targets have been explored in clinical practice.

### 4.1 Targeting MDM2: selective MDM2 inhibitors

A phase I study of the MDM2 inhibitor milademetan included 48 patients with recurrent or refractory WDLPS/DDLPS, with a median PFS (mPFS) of 6.3 months; one DDLPS patient achieved a partial response (69). The MANTRA study compared the efficacy of milademetan with that of trabectedin in 178 patients with unresectable or metastatic DDLPS who had failed to respond to prior treatments. No significant differences in mPFS were observed (3.6 months vs. 2.2 months, respectively), the median overall survival was comparable (9.5 months vs. 10.2 months, respectively), and the objective response rate did not significantly differ (4.7% vs. 3.4%, respectively) between the two treatments. On the basis of these findings, the MDM2 inhibitor failed as a second-line treatment for DDLPS (70).

### 4.2 Brigimadlin, an MDM2-p53 antagonist

A phase Ia study evaluated the efficacy of the MDM2-p53 antagonist brigimadlin in the treatment of 54 patients with advanced/metastatic MDM2-amplified and TP53 wild-type solid tumors. The overall objective response rate was 11.1% (6 of 54), the disease control rate was 74.1% (40 of 51), and the mPFS was 8.1 months. These findings indicate that brigimadlin has potential antitumor activity in patients with DDLPS and WDLPS. In the phase Ib (dose expansion) study, the number of evaluable DDLPS patients increased to 76 cases, with a preliminary mPFS of 8.1 months (95% confidence interval: 5.7–13.6 months), an objective response rate of 19%, and a disease control rate of 85%. Moreover, in the five evaluable WDLPS patients, the disease control rate was 100% (71). A phase II/III global multicenter study comparing brigimadlin with doxorubicin as first-line treatments for advanced DDLPS patients is currently underway (Clinical Trial: NCT05218499).

### 4.3 Targeting CDK4: CDK4/6 inhibitors

A phase II clinical study of palbociclib in 59 patients with WD/DDLPS revealed an mPFS of 17.9 weeks, with one patient achieving a complete response that lasted over 2 years. Thirty-six percent of the patients experienced grade 3–4 neutropenia (72). In 61 patients with retroperitoneal WDLPS/DDLPS treated with the single agent palbociclib, the practical application and surgical outcomes were as follows. The mPFS for WDLPS and DDLPS patients were 9.2 and 2.6 months, respectively. In addition, 12 patients ultimately underwent surgical resection, with half of the patients achieving R0/R1 resection; however, surgery did not improve overall survival (73).

Higuchi et al. reported that a combination of palbociclib and recombinant methioninase enhanced the efficacy of palbociclib against DDLPS in a patient-derived orthotopic xenograft mouse model of LPS (74). Moreover, a phase II clinical study of patients with recurrent or metastatic DDLPS treated with abemaciclib reported an mPFS of 30.4 weeks, with two patients achieving a partial response (75).

### 4.4 Combination of MDM2 inhibitors and CDK4/6 inhibitors

A phase Ib study combined siremadlin (a p53-MDM2 inhibitor) with ribociclib (a CDK4/6 inhibitor) in 74 patients with advanced WDLPS and DDLPS. Three patients achieved a partial response and 38 patients had stable disease, thus demonstrating the good antitumor activity of this combination treatment (76).

### 4.5 Targeting PARP1: PARP1 inhibitors

PARP1 expression is heterogeneous across subtypes. High PARP1 expression is mostly found in leiomyosarcoma, is often found in Grade 3 CINSARC (Complexity INdex in SARComas) and high-risk tumors, and is associated with a shorter MFS. By contrast, low PARP1 expression is mostly found in LPS and MFS (77). A multicenter, randomized, controlled phase II clinical study (TOMAS2) explored the efficacy of trabectedin combined with the PARP inhibitor olaparib versus trabectedin alone in 130 adult patients with STS whose previous treatments had failed. Of these, 67 patients had an L-sarcoma (LPS/leiomyosarcoma) subtype. The subgroup analysis did not yield positive results for mPFS or overall survival (78).

### 4.6 Targeting the nuclear export protein exportin 1

Zaffaroni et al. reported that selinexor (a selective XPO1 inhibitor) has stronger antitumor activity than doxorubicin against retroperitoneal DDLPS patient-derived xenografts (79). A phase Ib study of selinexor in the treatment of advanced STS included 15 DDLPS patients. Six patients experienced a reduction in the target lesion size and seven patients achieved stable disease as the best response; this was maintained for at least 4 months (80). A subsequent study, SEAL, included 285 patients with advanced DDLPS who had previously received two to three lines of treatment, and reported an mPFS of 2.8 months. The most common grade 3–4 adverse events associated with selinexor use were nausea (80.7%), decreased appetite (60.4%), and fatigue (51.3%) (81). Another study demonstrated that selinexor treatment can help to control pain and improve quality of life in patients with advanced DDLPS (82).

### 4.7 Targeting vascular endothelial growth factor

In the ALTER-0202 study, 13 patients with recurrent/metastatic advanced LPS were treated with anlotinib. This treatment resulted in a 12-week progression-free rate of 63%, and mPFS and median overall survival times of 5.6 and 13 months, respectively (83). The ALTER-S006 study revealed that anlotinib maintenance treatment resulted in an mPFS of 9.1 months in 49 STS patients who achieved a partial response or stable disease after at least four cycles of first-line anthracycline-based chemotherapy; LPS patients had an mPFS of 12.5 months (84). In another retrospective study, 17 patients with metastatic/recurrent WDLPS/DDLPS who were treated with anlotinib had an mPFS of 27.9 weeks, a 24-week progression-free rate of 58.8%, and an overall survival of 56.6 weeks (85). The aforementioned studies indicate the good efficacy of anlotinib for LPS, and the use of anlotinib as a second-line treatment for patients with STS is included in the Chinese Society of Clinical Oncology guidelines.

### 4.8 Multitarget tyrosine kinase inhibitors

The National Comprehensive Cancer Network guidelines recommend pazopanib as a second-line treatment option for patients with STS (86). In a phase II study, pazopanib was used to treat 41 patients with LPS (27 with DDLPS). This treatment resulted in a 12-week progression-free rate of 68.3%, and for DDLPS patients, the mPFS was 6.24 months (87). A multicenter phase II randomized controlled trial in Germany compared the efficacy of combined pazopanib and gemcitabine with pazopanib alone in the treatment of 90 patients with refractory STS (19% with LPS). There was a 12-week PFS of 74% vs. 47%, an mPFS of 5.6 months vs. 2.0 months, and an overall survival of 13.1 months vs. 11.2 months, respectively. However, the objective response rate was generally low, at 11% vs. 5%, respectively (88). Similarly, a previous study revealed that preoperative pazopanib treatment for nonmetastatic, resectable, high-risk STS did not benefit patients (89). In addition, in the SARC024 study, regorafenib treatment did not yield positive results for mPFS or overall survival in 48 patients with advanced LPS (90).

In summary, many types of targeted drugs have been used in the exploration of clinical treatments, and have achieved different results. Nonetheless, through continuous in-depth research, more accurate targets are expected to be obtained. The ultimate goal is to develop new drugs and novel solutions to improve the quality of life and survival of patients.

## 5 Research progress in immunotherapy for LPS

Multiple studies have shown broad heterogeneity in the tumor immune microenvironment of LPS based on tumor subtype, grade, size, multifocality, and primary or recurrent status (91, 92). Regarding immune microenvironments, research has mainly focused on DDLPS and MLPS; WDLPS and PLPS are therefore less understood. DDLPS is characterized by a greater abundance of TIL and a higher expression of PD-L1, whereas MLPS displays the opposite characteristics, and WDLPS is likely positioned between the two (93, 94). In terms of treatment, immune checkpoint inhibitors, therapeutic antibodies, and tumor vaccines (95), immunomodulators (96), adoptive cell therapy, and T-cell receptor−genetically engineered T-cells may become new options for patients with advanced unresectable LPS. At present, the initial efficacy of immune checkpoint inhibitor monotherapy in LPS patients is poor (97). Nonetheless, the combination of immune checkpoint inhibitors with other strategies—such as chemotherapy (98), VEGF blockers (99), cytokines, immunomodulators, radiotherapy, and other regimens—is being actively explored, and is expected to improve the oncological prognosis of LPS.

## 6 Discussion

With the rapid development of medical science and technology and the continuous innovation of research methods, important progress has been made in the research and treatment of LPS. Clinical studies related to LPS targeted therapy have been collected by the authors and presented in table form. Information on clinical trials that have been completed can be found in **Table 1**. Information on ongoing clinical trials can be found in **Table 2**. In terms of LPS occurrence and the mechanisms of LPS development, extensive heterogeneity and unique characteristics exist at the molecular level. Notably, the rapid development of molecular diagnostic technology is opening the door to understanding these molecular mechanisms in a stepwise manner. Moreover, “targeted therapy” has been launched at the molecular level.

**Table 1:** Clinical trials related to LPS-targeted therapy.

Clinical trial	Medication regimen	Pathway target	Phase	Objective	Primary endpoint	Secondary endpoints	Primary outcome	Author	Year of publication	References
NCT01877382	DS-3032b	Disrupts the MDM2-p53 interaction	I	94 pts: WD/DD LPS (40, 43%)	MTD, PK,PD, efficacy	–	47(60%)SD; Median duration of SD:6.7 (1.6 to 36.4) m; 1 PR in DDLPS.	Todd Michael Bauer	2018	(69)
MANTRA (RAIN-3201; NCT04979442)	Trabectedin+milademetan vs. Trabectedin	A selective, potent, small molecule inhibitor of the MDM2-p53 interaction	III	175 DDLPS pts	PFS	OS; ORR	M-follow-up:2.1m (range 0–13m). mPFS was numerically higher. mOS and ORR was comparable in the two arms.	R.L. Jones	2023	(70)
NCT03449381	Brigimadlin (BI 907828)	An oral MDM2–p53 antagonist	Ia	28 (51.9%) sts pts: 12 DDLPS and 7 WDLPS	MTD、Pk、Pd、Activity	–	WD/DDLPS ORR:100%; WD/DDLPS DCR:75%; DDLPS with duration of SD 1.5∼22 m	Patricia LoRusso	2023	(71)
NCT01209598	Palbociclib	The selective CDK4 and CDK6 inhibitor	II	60 WD/DDLS pts	PFS	PFR12 weeks	PFR12 weeks:57.2%; mPFS:17.9 weeks; 1 CR; Toxic effects:hematologic、neutropenia.	Mark A. Dickson, MD	2016	(72)
NCT02846987	abemaciclib	A newer and more potent CDK4 inhibitor	II	30 DDLS pts	PFR12 weeks	–	PFR12 weeks:76%; mPFS:30.4 weeks; one PR; Grade 3-4 toxicity:anemia、neutropenia、thrombocytopenia、diarrhea.	Mark Andrew Dickson	2019	(75)
NCT02343172	Siremadlin+ ribociclib	A p53–MDM2 inhibitor;a CDK4/6 inhibitor	Ib	74 WDLPS or DDLPS pts	MTD、RDE	–	RDE: siremadlin 120 mg every 3 weeks+ribociclib 200 mg QD; 3 PR, 38 SD.	Albiruni R. Abdul Razak	2022	(76)
TOMAS2(NCT03838744)	Trabectedin+Olaparib	PARP1 Inhibitor	II	130 STS pts (L–sarcoma 67)	PFS6m	PFS, OS, ORR, DOR, safety.	m-follow-up:10.2 mo; T+O PFS6mo:32%; T+O mPFS:4.0 mo; T+O PFS12mo:20%	L D’Ambrosio	2023	(78)
NCT01607905	selinexor	Selective inhibitor of nuclear export compound	Ib	54 sts pts (WD/DD 16;MLS 3)	PK,PD,efficacy	–	none ORR; SD (≥ 4 months):17 (33%), including 7 (47%) DDLPS.	Mrinal M. Gounder	2016	(80)
SEAL(NCT02606461)	S (Selinexor) vs.placebo (P)	Selective inhibitor of nuclear export compound	II/III	51 DDLS pts	PFS by WHO	–	PFS (WHO):no difference; mPFS (R v1.1): 5.6 mo vs.1.8 mo, hr0.64. Grade 3/4 AEs: hyponatremia, anemia, thrombocytopeniam	Mrinal M. Gounder	2018	(81)
ALTER-0202 (NCT01878448)	Anlotinib	A multikinase angiogenesis inhibitor	II	166 STS pts , LPS (n = 13)	PFR12 weeks	–	LPS PFR 12weeks:63% LPS mPFS:5.6m LPS mOS:13 m LPS ORR:7.7%	Yihebali Chi	2018	(83)
ALTER-S006(NCT03890068)	Anlotinib as a maintenance treatment	multikinase angiogenesis inhibitor	II	49 STS pts, 17 lps (35%)	PFS	OS, ORR, DCR and safety.	M-follow-up:17.1 mo; mPFS:9.1 mo; LPS mPFS:12.5 mo(95% CI 7.1–18.0); WDLS (n= 4) and DDLS (n = 11) mPFS:19.1 mo、9.0 mo; Overall ORR and DCR:16%(8/49)、94% (46/49); Lps ORRs and DCRs:12% (2/17)、 100% (17/17) ; WDLS ORRs and DCRs:25%(1/4)、100%; DDLS ORRs and DCR:9% (1/11)、100%; MLPS:2 SD, DCR 100%.	Bushu Xu	2023	(84)
EORTC 62043	Pazopanib	multityrosine kinase	II	142 STS pts:LPS (19)	PFR12 weeks	response, safety,os	LPS PFSR 12 weeks :26% (5/19); LPS mPFS:80 d; LPS mOS:197; AEs:hypertension, fatigue, hypopigmentation, nausea	Stefan Sleijfer	2009	(100)
NCT01506596	Pazopanib	multitargeted TKI with activity against VEGF and PDG	II	41 lps pts:DDLPS 27 (65.9) ;MLS 12 (29.3) ;PLPS 2 (4.9)	PFR12 weeks	–	PFR12:68.3%; mPFS:4.4mo; mOS:12.6mo; AEs:nausea (39%), hypertension (36.6%), diarrhea (34.1%), and fatigue (29.3%)	Brian L. Samuels	2017	(87)
DRKS00003139	Pazopanib+gemcitabine (A) vs. gemcitabine (B)	multityrosine kinase	II	90 STS pts:LPS (16, 19%).	PFSR 12 weeks	Toxicity, os, ORR	M-follow-up:12.4mo;PFSR 12 weeks :74% (A) vs 47% (B) (HR, 1.60; P = .01). mPFS:5.6 vs 2.0 mo(HR, 0.58;P = .02) mOS:13.1 vs 11.2 mo(HR, 0.98; P = .83). ORR:11% (A) vs 5% (B) (P = .10).	Hans-Joachim Schmoll	2021	(88)
NCT01900743	Regorafenib vs. placebo	A multitargeted kinase inhibitor with a kinase profile overlapping	II	37 STS pts:LPS (20)	PFS	OS	mPFS:1.1m; mOS:4.7m; ORR:0%	Nicolas Penel	2019	(101)
SARC024 (NCT02048371)	Regorafenib vs. placebo	A multitargeted kinase inhibitor with a kinase profile overlapping	II	48 LPS pts(34 DDLPS, 12 MLPS, 2 PLPS)	PFS	–	mPFS:1.87 mo vs. 2.07mo, HR=0.85, p = .62. No responses on regorafenib. mOS:6.46mo vs. 4.89mo, HR=0.66, p = .28.	Richard F. Riedel	2023	(102)

**Table 2:** Clinical trials of LPS-targeted and immunotherapy are currently underway.

Regisition number	Pathway target	Study Status	Phase	Objective	Intervention	Primary outcome	Location	Years study Started
NCT06414434	a type of kinase inhibitor	RECRUITING	I	LPS	DRUG: BTX-A51	Safety and tolerability	America	2024/9/1
NCT06389799	combination of an FGFR inhibitor, pemigatinib, with a PD-1 inhibitor, retifanlimab	RECRUITING	II	advanced DDLPS	DRUG: Pemigatinib、Retifanlimab	OS、ORR	Europe	2024/6/20
NCT05580588	the CDK4/6 Inhibitor, a selective enzyme blocker	RECRUITING	II	Locally Advanced or Metastatic LPS	DRUG: SPH4336	PFS at 12 weeks	America	2023/8/31
NCT05496569	the CDK4/6 Inhibitor	NOT_YET_RECRUITING	II	DDLPS	DRUG: TQB3616 capsule	PFS	China	2022/12/1
NCT06058793	a so-called MDM2 inhibitor	ACTIVE_NOT_RECRUITING	III	DDLPS	DRUG: Brigimadlin(BI 907828)	TEAEs	America	2023/12/12
NCT05218499	a so-called MDM2 inhibitor	ACTIVE_NOT_RECRUITING	II/III	DDLPS	DRUG: Brigimadlin (BI 907828)、Doxorubicin	PFS	America	2022/3/25
NCT00969917	HSP90 inhibitor	WITHDRAWN	II	DDLPS	DRUG: IPI-504	safety profile and ORR	America	2010/1/1
NCT04967521 (SARC041)	the CDK4/6 Inhibitor	RECRUITING 108	III	DDLPS	DRUG: Abemaciclib、Placebo	PFS	America	2021/11/11
NCT02846987	the CDK4/6 Inhibitor	ACTIVE_NOT_RECRUITING	II	DDLPS	DRUG: Abemaciclib	PFS	America	2016/7/1
NCT05886634	the A2AR/A2BR Inhibitor;PD-1 Inhibitor	RECRUITING	II	DDLS	DRUG: Etrumadenant、Zimberelimab	ORR	America	2023/5/23
NCT03074318	Immunotherapy with monoclonal antibodies、chemotherapy	TERMINATED	I/II	Liposarcoma or Leiomyosarcoma	DRUG: Avelumab、Trabectedin	Adverse Events, ORR	America	2017/9/28
NCT04785196	APG-115 Combination With PD-1 Inhibitor(toripalimab)	RECRUITING	Ib/II	Advanced LPS or Advanced Solid Tumors	DRUG: APG-115、 Toripalimab	Dose Limiting Toxicity (DLT)、MTD、ORR	Britain	2021/5/26
NCT04438824	PD-1 Inhibitor;the CDK4/6 Inhibitor	ACTIVE_NOT_RECRUITING	II	DRUG: INCMGA00012、Palbociclib	Advanced LPS	RP2D、DLTs、orr	America	2020/6/17
NCT02571829	the CDK4/6 Inhibitor	–	II	DRUG: ribociclib	Advanced WD/DDLPS	Response	Jerusalem、Israel	2016/5/1
NCT03096912	the CDK4/6 Inhibitor	–	II	DRUG: Ribociclib	Advanced WD/DDLPS	Response	Be’er Ya’aqov,Israel	2016/7/1
NCT02587169	the BCR-ABL Inhibitor	–	I/II	DRUG: Nilotinib、adriamycin	LPS and LMS of Retroperitoneum	RFS at 5 years、OS	Spain	2012/1/1
NCT03307616	Immunotherapy with monoclonal antibodies	ACTIVE_NOT_RECRUITING Ii 32	II	Ipilimumab、Nivolumab、Radiation therapy when given before surgery works	undifferentiated pleomorphic sarcoma or ddlps	Pathologic response	America	2017/10/4
NCT05694871	blocking some of the enzymes;PD-1 Inhibitor	RECRUITING	II	DRUG: Palbociclib、Cemiplimab	Advanced Dedifferentiated Liposarcoma	PFS;Efficacy analyses; PFS rates at 12, 24, 36, and 48 months	America	2023/5/30
NCT01876043	–	TERMINATED	II	DRUG: plitidepsin	Advanced Unresectable or Metastatic, Relapsed/Refractory, DLPS	Percentage of Patients Remaining Alive and Progression Free at 3 Months	France	2012/2/1
NCT02609984	Vaccine;PD-L1 Inhibitor	TERMINATED	II	CMB305、atezolizumab	sarcoma (synovial or mrlps) expressing the NY-ESO-1 protein	PFS、OS	America	2015/4/29
NCT03114527	the CDK4/6 Inhibitor;the mTOR Inhibitor	ACTIVE_NOT_RECRUITING	II	DRUG: Ribociclib、Everolimus	advanced LMS or DDL	Tumor response、Progression free rate, 16 weeks	America	2017/8/8
NCT04356872	PD-1 immune check point inhibitor, sintilimab, in combination of stand of care chemotherapy	–	II	DRUG: Sintilimab、Doxorubicin 、Hydrochloride、Ifosfamide	Select Type of Metastatic/Unresectable Soft Tissue Sarcoma	ORR	China	2020/4/8
NCT02059850	Vaccin	WITHDRAWN	I	Autologous NY-ESO-1-specific CD8-positive T Lymphocytes、Cyclophosphamide	advanced synovial sarcoma or myxoid/round cell liposarcoma.	toxicity	America	2014/7/1
NCT06498648	kinase inhibitors	NOT_YET_RECRUITING	1/2	DRUG: Abemaciclib、Docetaxel、Gemcitabine	STS(LMS、DDLPS)	Time course of blood thymidine kinase activity (TKa)		2024/9/30
NCT04242238	the CSF1R Inhibitor;PD-L1 Inhibitor	ACTIVE_NOT_RECRUITING	I	DRUG: DCC-3014、Avelumab	Advanced or Metastatic Sarcomas (DDLPS)	MTD、ORR	America	2020/1/22
NCT03880123	the XOP1 Inhibitor	WITHDRAWN	I	DRUG: Selinexor、Ixazomib	Advanced Sarcoma	MTD、ORR	America	2020/11/1
NCT06116578	Immunotherapy、 DNA repair inhibitor	NOT_YET_RECRUITING	II	DRUG: Pembrolizumab、 Olaparib	tertiary lymphoid structures (TLS) Positive Selected Resectable STS	Rate of CD8+ T-cell tumor infiltration density at surgery compare to baseline	France	2024/9/1
NCT05813327	–	RECRUITING	I/II	DRUG: Neoadjuvant ADI PEG20、Ifosfamide、Radiotherapy、Mesna	STS	SAE rate、RP2D、efficacy	America	2024/3/14
NCT05497843	kinase inhibitors		II	DRUG: Chiauranib	Advanced or Unresectable STS	PFS12w	China	2022/8/2
NCT03064243	a kinase inhibitor of receptor tyrosine with VEGFR2.	–	II	DRUG: apatinib	STS	6 months PFS rate	China	2017/3/1
NCT01878448	a kinase inhibitor of receptor tyrosine with multi-targets, especially for VEGFR2 and VEGFR3.	COMPLETED	II	DRUG: Anlotinib	STS	effectiveness	China	2017/4/30
NCT03121846	TKI		II	DRUG: Apatinib	PFS	STS	China	2017/5/1
NCT02449343 (ALTER0203)	TKI		II/III	DRUG: Anlotinib、Placebo	PFS	STS	China	2015/5/12
NCT00276302	HSP90 inhibitor	COMPLETED	I	IPI-504	safety and MTD of IPI-504 in GIST and STS	GIST or STS	America	2005/12/1
NCT03217266	blocking some of the enzymes needed for cell growth	ACTIVE_NOT_RECRUITING	Ib	DRUG: Navtemadlin、Radiation Therapy	STS	Mtd	America	2018/6/20
NCT01995981	multitargeted TKI with activity against VEGF and PDG	COMPLETED	IV	DRUG: Pazopanib	STS	evaluate whether early metabolic response is correlated to clinical benefit:FDG uptake	Britain	2013/12/1
NCT02636725	multitargeted TKI with activity against VEGF1-3;PD-1 Inhibitor	COMPLETED	II	DRUG: Axitinib、Pembrolizumab	STS	PFS at 3 Months	America	2016/4/19
NCT01418001	TKI	TERMINATED	Ib/II	DRUG: Neoadjuvant Pazopanib、Gemcitabine、Docetaxel	STS	ORR	America	2011/8/1
NCT05926700	PD-1/CTLA-4 Inhibitor	RECRUITING	II	DRUG: Candonilimab	Advanced STS	ORR	China	2024/2/28
NCT03138161	Immunotherapy	RECRUITING	I/II	DRUG: Trabectedin、Ipilimumab、Nivolumab	Advanced STS	MTD	America	2017/4/13
NCT05448820	CTLA-4 Inhibitor;PD-L1 Inhibitor	ACTIVE_NOT_RECRUITING	I/II	DRUG: YH001、Envafolimab、Doxorubicin	Advanced or Metastatic Sarcoma	RP2D、ORR	America	2022/11/14
NCT02451943	A PDGFRa Inhibitor	COMPLETED	III	DRUG: Olaratumab、Doxorubicin、Placebo	Advanced or Metastatic STS	OS	America	2015/9/14
NCT01975519	Anti-endoglin antibody	COMPLETED	I/II	DRUG: TRC105 and Pazopanib	Advanced STS	RP2D、PFS、DLT、ORR	America	2013/12/10
NCT00626704	a DR5 agonistic antibody	COMPLETED	I/II	DRUG: AMG 655、Placebo、Doxorubicin	Unresectable STS	PFS	–	2007/11/1

Ongoing research into LPS has led to important advancements in understanding its molecular biology. The identification of numerous genes, their RNA products, and associated downstream pathways has presented many potential targets for therapeutic intervention. In the future, the development of targeted treatment strategies based on these insights will be paramount.

Given the diversity of STS subtypes and their relatively low incidence compared with other malignancies, the research community faces the challenge of addressing a “rare” tumor with a dispersed pathology. The trajectory of future LPS research should therefore focus on two main avenues: identifying specific histological subtypes to reveal subtype-specific therapeutic opportunities, and discovering precise biomarkers to identify patient populations that are most likely to benefit from targeted therapies. Personalized medicine—crafted according to the intricate interplay of histological and molecular profiles—holds great promise for the treatment of LPS.

The progress made thus far lays a solid foundation for the next steps in LPS research. As we continue to unravel the complexities of this disease, the integration of molecular insights with clinical practice will be essential. Through this collaborative and targeted approach, we hope to improve the outcomes for patients with LPS.
